# Development of an early alert model for pandemic situations in Germany

**DOI:** 10.1038/s41598-023-48096-3

**Published:** 2023-11-27

**Authors:** Danqi Wang, Manuel Lentzen, Jonas Botz, Diego Valderrama, Lucille Deplante, Jules Perrio, Marie Génin, Edward Thommes, Laurent Coudeville, Holger Fröhlich

**Affiliations:** 1 Department of Bioinformatics, Fraunhofer Institute for Algorithms and Scientific Computing (SCAI), Schloss Birlinghoven, 53757 Sankt Augustin, Germany; 2 Bonn-Aachen International Center for IT, University of Bonn, Friedrich Hirzebruch-Allee 6, 53115 Bonn, Germany; 3 Quinten Health, 8 Rue Vernier, 75017 Paris, France; 4 Sanofi, Paris, France

**Keywords:** Health care, Scientific data

## Abstract

The COVID-19 pandemic has pointed out the need for new technical approaches to increase the preparedness of healthcare systems. One important measure is to develop innovative early warning systems. Along those lines, we first compiled a corpus of relevant COVID-19 related symptoms with the help of a disease ontology, text mining and statistical analysis. Subsequently, we applied statistical and machine learning (ML) techniques to time series data of symptom related Google searches and tweets spanning the time period from March 2020 to June 2022. In conclusion, we found that a long-short-term memory (LSTM) jointly trained on COVID-19 symptoms related Google Trends and Twitter data was able to accurately forecast up-trends in classical surveillance data (confirmed cases and hospitalization rates) 14 days ahead. In both cases, F1 scores were above 98% and 97%, respectively, hence demonstrating the potential of using digital traces for building an early alert system for pandemics in Germany.

## Introduction

Coronavirus disease 2019 (COVID-19) is an infectious disease caused by the severe acute respiratory syndrome coronavirus type 2 (SARS-CoV-2) that emerged in December 2019. It was discovered in Wuhan, China, and quickly spread over the world, including Europe and the United States. In early 2020, almost three million positive cases were identified worldwide^1^. At the time of the first outbreak in China, many healthcare systems around the world were not well prepared for the pandemic. Measures to prevent its spread to other regions of the world were often hesitant and taken too late. In the light of this situation one of the missions of the French-German collaborative project AIOLOS (Artificial Intelligence Tools for Outbreak Detection and Response (https://aiolos-project.org)) is to implement modeling approaches that could support the development of an early warning system for pandemic situations.

Public health surveillance data, such as confirmed cases, hospitalizations and deaths, are critical for understanding disease epidemiology^2^. One big challenge, though, is that many countries do not always record traditional surveillance data in a fully automated and digital way in real time. Hence, there is a systematic and critical delay between reported surveillance and the real disease spread. However, time is a critical element for taking effective countermeasures against epidemic or pandemic situations^3,4^. In response to this situation, Westhaus et al. proposed systematic wastewater monitoring^5^, and according measures have been implemented in the USA, Australia, Israel, Spain, Italy and several French regions during the COVID-19 pandemic^6–15^. However, wastewater monitoring is expensive and requires knowledge of the specific virus particles that are searched for.

A principle alternative is the use of social media and other digital traces of people’s online activities as a source of disease associated information. The underlying hypothesis is that people tend to search for signs of illness and post about them in social networks before confirmed cases are reported in traditional surveillance data. Hence, information may spread faster through digital traces compared to traditional channels. Over the last decade, several studies have explored this idea, for example, by leveraging Google searches and Twitter posts for predicting and tracking the spread of diseases such as the flu, dengue, Zika, MERS, Ebola and COVID-19^2,16–22^. We refer to Botz et al.^23^ for a more detailed review. For example, Kogan et al. used a Bayesian model to develop an early warning algorithm for COVID-19 based on several data sources (Google Trends, Twitter, UpToDate), fever incidence rates, and predictions made by the global epidemic and mobility model in the USA^2^. The algorithm was validated on US COVID-19 surveillance data as well as incidence rates of influenza-like illness, demonstrating that an uptrend in COVID-19 infections could be predicted up to 7 days in advance with a sensitivity of 0.75. Noteworthy, conclusions made in this paper cannot necessarily be generalized to other regions of the world, because the use of digital platforms may display socio-economical and cultural differences. To the best of our knowledge, no comparable study has been conducted for Germany thus far.

In this work we tried to fill this gap by developing a neural network (LSTM) based machine learning model to predict trends in surveillance data using Google Trends and Twitter data for Germany from March 2020 to June 2022. In particular, our work demonstrates the LSTM model built on the combined data traces has the possibility to forecast up-trends in COVID-19 case and hospitalization numbers 14 days in advance with sensitivity values of 0.96 and 1, and F1 scores of 0.98 and 0.97, respectively.

Our work thus highlights the applicability of deep learning models for COVID-19 forecasts and demonstrates that digital traces, such as Google Trends and Twitter, could be useful sources for developing an early warning system for pandemic outbreaks in Germany.

## Methods

### Framework overview

**Figure 1:**
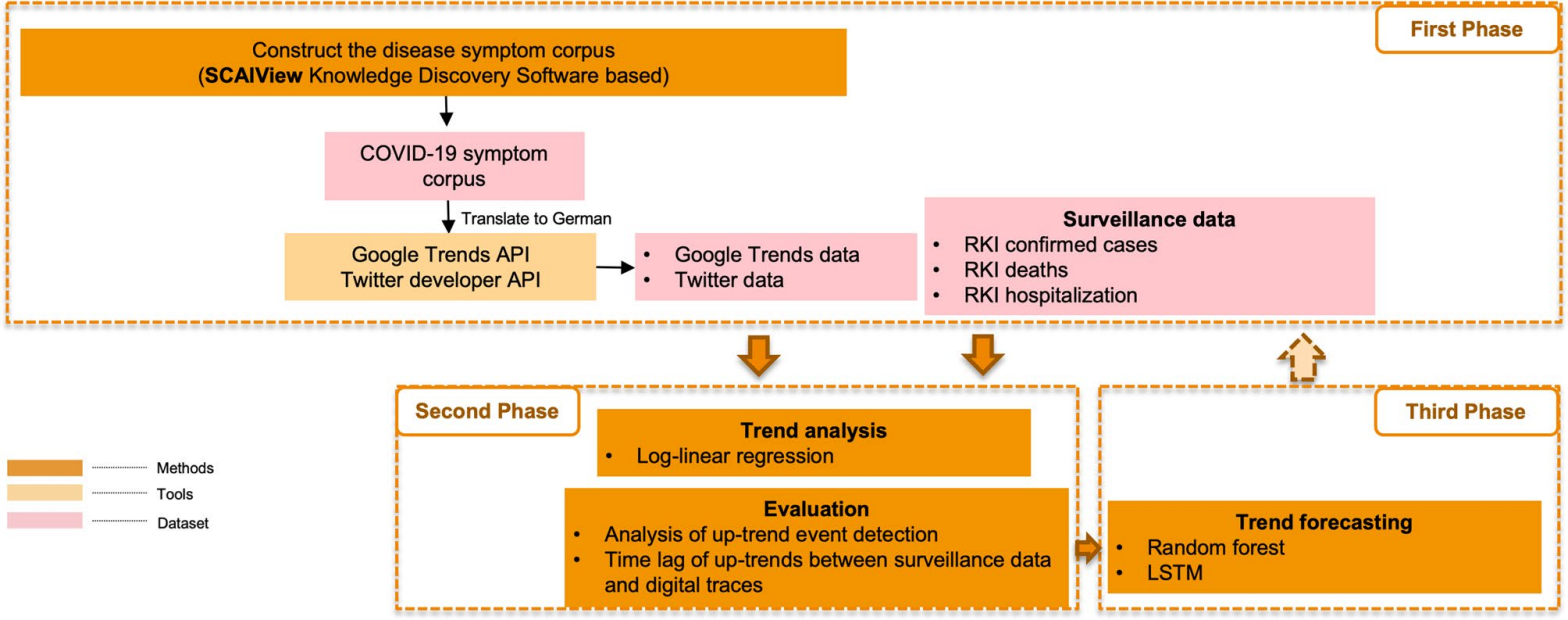
The proposed workflow of developing an early alert model using digital traces from Google Trends and Twitter.

This study presents a three-phase framework for developing early warning models (Fig. 1). Initially, a German disease symptom corpus was created via ontology and text mining as well as statistical analysis. This was followed by data collection from Google Trends and Twitter using their respective APIs. In the second step, we examined the relationship between trends in digital traces and surveillance data using a log-linear regression model. Lastly, trend forecasting models (Random Forest and LSTM) were developed.

### Disease symptom corpus

A literature-based text-mining approach for identifying COVID-19 related symptoms was implemented. For that purpose, we first downloaded 968 symptoms and their 1209 synonyms from the EBI Symptom Ontology (https://www.ebi.ac.uk/ols/ontologies/symp, accessed on June 2, 2022), and searched those symptoms as well as COVID-19 related terms in SCAIView knowledge discovery software (https://academia.scaiview.com). SCAIView allows semantic searches in full-text biomedical articles (PubMed and PMC) by combining free text searches with the ontological representations of automatic recognized biological entities^24–26^. The search term body was generated as (1) a respective symptom or synonym, (2) COVID-19-related terms (“COVID”, “Coronavirus disease”, “COVID 19”), (3) both together. We then retrieved the number of corresponding documents via SCAIView Academia REST-API (https://api.academia.scaiview.com/swagger-ui.html, accessed on June 15, 2022).

To narrow down the extensive list of potential search terms, we aimed to identify the most significant ones by assessing their frequency of appearance in PubMed and PMC articles compared to what would be expected by chance. To accomplish this, we conducted a hypergeometric test for each term^27^. The test was performed as follows: Let *M* be the total number of documents in SCAIView search engine, and *n* be the number of documents containing COVID-19-related terms. Furthermore, let *N* be the number of documents that contain a certain symptom/ synonym *q*. Finally, let *x* be the number of documents that contain both, COVID-19-related terms and *q*, i.e. the cardinality of the intersection of both sets. Then the probability of seeing an intersection at least as large as *x* is given by

1 $$\begin{aligned} \textit{P}(X\ge x)=1-\sum \nolimits _{i=0}^{x-1}\frac{\left( {\begin{array}{c}n\\ i\end{array}}\right) \left( {\begin{array}{c}M-n\\ N-i\end{array}}\right) }{\left( {\begin{array}{c}M\\ N\end{array}}\right) } \end{aligned}$$

Holm–Bonferroni method was used to correct for *P* values of all symptoms for multiple testing^28^. For generating the final symptom corpus, all symptoms were translated into German.

### Google trends

Google allows all users to access and process anonymized data on relative search volume behavior with Google Trends. Using the Google Trends API, we obtained a daily time series of the search frequencies for the German symptom keyword through the Pytrends library (version 4.9.0). The TrendReq method was used to pass host language (“de-DE”) and timezone offset (60) in the initialization step. We queried each symptom keyword term in a measure entitled “interest_over_time”. Parameters such as “start_year” (2020), “start_mon” (1), “stop_year” (2022), “stop_mon” (6), and “geo” (“DE”) were given in the function. The relative search result for a given keyword is between 0 and 100. A value of 0 indicates the lowest relative search interest for the given keyword, whereas a value of 100 indicates the date with the maximum search interest. For consistency, the daily data values are scaled by multiplying the daily value by the number of searches per month divided by 100 (https://github.com/GeneralMills/pytrends/tree/master/pytrends, accessed on June 15, 2022). The Google Trends dataset includes data from January 5, 2020 to June 28, 2022. We eliminated symptoms which consistently showed no Google queries throughout the entire time span.

### Twitter microblogs

The study leveraged Twitter’s streaming API and Tweepy library (version 4.8.0) to collect Twitter data. The streaming API allows for a stream of public tweets from the platform in real time that can be filtered using multiple parameters. Credentials of Academic Developer Portal are applied for getting the full-archive daily tweets counts using “get_all_tweets_count” in the case of a client application with the support of Pagination. Pagination programmatically retrieves the entire result set by setting “flatten” with a larger limit. In our study, each German symptom keyword and language (“DE”) were specified at the query part. The Twitter dataset contains the daily search amount for certain German symptoms between January 1, 2020 and June 28, 2022. Similar to the situation with Google Trends, we removed symptoms for which no tweets could be found.

### Surveillance data

In this study, we obtained daily COVID-19 case reports from Robert Koch-Institute (RKI) website as the ground-truth, including RKI confirmed cases, RKI deaths (https://github.com/robert-koch-institut, accessed on March 24, 2023), and RKI hospitalization (https://github.com/robert-koch-institut/COVID-19-Hospitalisierungen_in_Deutschland, accessed on February 22, 2023). The time series of surveillance data started on March 1, 2020, and we sliced it till June 28, 2022 for further analysis. Overall, it provided insight into the changing trends and patterns of the monitored area over a two and a half year period. Data has been normalized based on the number of corresponding cases per 100,000 people.

### Statistical trend analysis

#### Trend decomposition

The occurrence of symptom mentioning in social media and Google searches may be confounded by natural seasonal fluctuations, resulting in an elevated level of false positive alarms.To remove unwanted such fluctuations, we thus applied robust time series decomposition techniques, more specifically Seasonal and Trend decomposition using LOESS (STL), a non-parametric method developed by Cleveland et al.^29^. STL has the strengths of simplicity and effectiveness when dealing with time series data as each component is allowed to change over time, and the system is robust to outliers^30^.

STL is a filtering procedure which is used for decomposing a seasonal time series into three components, which are called trend, seasonal, and remainder. The supposed raw data, the trend component, the so-called seasonal component, and the remainder component are denoted by $Y_v$, $T_v$, $S_v$, and $R_v$, respectively. For *v* = 1 to N:

2 $$\begin{aligned} Y_v= T_v+S_v+R_v \end{aligned}$$

STL decomposition consists the following two recursive procedures: an inner loop and an outer loop. The inner loop updates the seasonal and trend components with seasonal smoothing and trend smoothing, which is handled by locally-weighted regression (LOESS)^31^. The procedure consists of six iterative steps as follows^32^: (1) Detrending: The detrended time series can be calculated via the formula $y_{v}-{T_v}^{(k)}$, where *k* denotes the iteration. (2) Cycle-Subseries Smoothing: To smooth the subseries of detrended data and obtain the smoothing result, which is shown as $C_v^{(k+1)}$. (3) Low-Pass Filtering: Two moving average filters and a LOESS smoother are used in this procedure. The result is shown as $L_v^{(k+1)}$. (4) Detrending of Smoothed Cycle-Subseries is performed to get the seasonal component: $S_v^{(k+1)} = C_v^{(k+1)}-L_v^{(k+1)}$. (5) De-seasonalizing: Subtract the seasonal component. $y_v-S_v^{(k+1)}$. (6) Trend Smoothing: LOESS is used to smooth the deseasonalized series and to get the trend component of current pass: $T_v^{(k+1)}$.

In the outer loop a robustness weight ($\rho _{v}$) is defined, which reflects how extreme the remainder component is weighted: $\rho _{v} = B(|R_{v}|h)$, where $h=6\times median(|R_{v}|)$ and B is the bisquare function:

$$ B(u) = \left\{ {\begin{array}{*{20}l}    {(1 - u^{2} )^{2} } \hfill & {0 \le u < 1} \hfill  \\    0 \hfill & {u > 1.} \hfill  \\   \end{array} } \right. $$

$\rho _{v}$ is adjusted to reduce the influence of outliers in Cycle-Subseries and Trend Smoothing. We used the “STL” function in Python statsmodels library (version 0.13.5) to decompose the raw time series data, and “period” parameter was specified to adjust the seasonal part. For surveillance data, the “period” parameter was set to 7, and for digital traces time series data, the value was 30.

#### Log-linear regression model

We assume that pandemic events exhibit exponential behavior within a relatively short time frame. Our study thus employs a log-linear regression model to track the growth and decline of surveillance and digital traces in consecutive 14-day intervals throughout the training period, which spans from February 2020 to February 2022. This duration encompasses various phases of the pandemic.

3 $$\begin{aligned} \begin{array}{l} \log (Y_t) = \alpha + \beta * t + \varepsilon _t \\ \varepsilon _t\sim N(0, \sigma ^{2}) \end{array} \end{aligned}$$

In Eq. 3, the independent variable is the “time”, more specifically, the days in our case, $\alpha$ is the estimated intercept, $\beta$ is the slop coefficient, $\varepsilon _t$ represents the Gaussian noise term with a mean of 0 and a variance of $\sigma^2$, and $Y_t$ represents the trend component at time *t*, which has previously been extracted via STL decomposition. The log transformed surveillance and digital time series data is fitted against days over the respective 14-days interval (sliding window).

**Figure 2:**
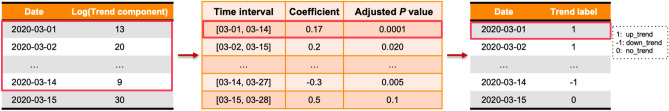
Illustration of fitting the log-linear regression model and identifying the corresponding trend of each date. Surveillance and digital traces data is fitted within a successive 14-days time interval (sliding window). For each sliding window, the statistically significant coefficient and adjusted *P* values are calculated, e.g. An “up-trend” (1) is given for the sliding window as the coefficient $\beta$ is larger than 0 and the adjusted *P* value is less than 0.05 threshold.

We evaluated the statistical significance of coefficient $\beta$ within each sliding window followed by an adjustment to *P* values using the Holm–Bonferroni method (5 % significance level), i.e. $\log (Y_{t_0:t_{13}})$ was the first window. We determined the trend given $\beta$ and the respective *P* value for every interval. When $\beta$ exceeded 0 and the adjusted *P* value was less than 0.05, an exponential growth (“up-trend”) was declared, whereas an exponential decay (“down-trend”) was defined when $\beta$ was less than 0 and the adjusted *P* value less than 0.05. For other situations, we declared “no trend”. This procedure was repeated on successive days to obtain a sequence of adjusted *P* values and trends. The illustration of the log-linear regression model is shown in Fig. 2. For subsequent analysis we always assigned $\beta$ to the first time point of the sliding window. Since the sliding window has a stride of 1 day, we were thus able to convert the original time series into a time series of $\beta$ coefficients (i.e. slopes), one per day, except for the last 13 days.

#### Multi-symptom *P* value

To obtain a comprehensive metric for combining multiple symptoms of each digital data trace, the harmonic *P* (HMP) value approach was utilized, as described in study^2,33^. The calculation involved combining *P* values estimated for each symptom/synonym. The formula used for this calculation is as follows:

4 $$\begin{aligned} \textit{P} = \frac{ {\textstyle \sum _{i=0}^{k}}w_i}{{\textstyle \sum _{i=0}^{k}}w_iP_i^{-1}} \end{aligned}$$

where $w_i$ were weights that sum to 1, and each symptom/synonym was treated equally. Following that, *P* values were adjusted for multiple testing using the Holm–Bonferroni method at a significance level of 5%^28^. In particular, we voted for the trend across multiple symptoms per day, i.e. an “up-trend” was declared only if the majority of symptoms demonstrated an up-trend and the adjusted *P* value was less than 0.05. We assigned a “down-trend” using the same strategy, otherwise a “no-trend” was declared.

In case of harmonizing digital traces, the metric was calculated by combining the *P* value estimates across Google Trends and Twitter.

#### Evaluating early alerts in digital traces

To quantify the performance of digital traces as potential early indicators of the onsets of up-trends in surveillance data, we defined the true-positive rates (TPR), false-positive rates (FPR) and false-negative rates (FNR) of the up-trends in agreement to Kogan et al.^2^. Note that the evaluation metrics were calculated during February 2020 to February 2022, and only the first detected up-trend in digital traces and surveillance data was considered when the same trend occurred for a period of time before a different event (down- or no-trends) was observed. (1) A true-positive (TP) event was declared, if an up-trend in the digital data source (an alert) fell within a 30 days window ahead of the onset of up-trends detected in surveillance data (confirmed cases, hospitalization, and deaths). The 30 days were counted from the beginning of each sliding window. (2) A false-negative (FN) event was declared, if an up-trend onset was detected in the surveillance data, but no alert was found in the digital data source within the 30 days time window ahead of this event. (3) A false-positive (FP) was declared, if an alert in the digital data source was found, but no up-trend was detected within a 30 days time window in the surveillance data afterwards.

Given these definitions, sensitivity, precision, and F1 score could be calculated. More specifically, the sensitivity is the ratio between the TP events (“successful alerts”) and the sum of TP and FN events (the number of up-trend onsets in the surveillance gold standard), and the precision is the ratio between TP events and the sum of TP and FP events (the number of alerts observed in digital traces). Evaluation metrics for down-trends can be calculated in a likewise manner.

### Trend forecasting via machine learning

#### General setup

**Figure 3:**
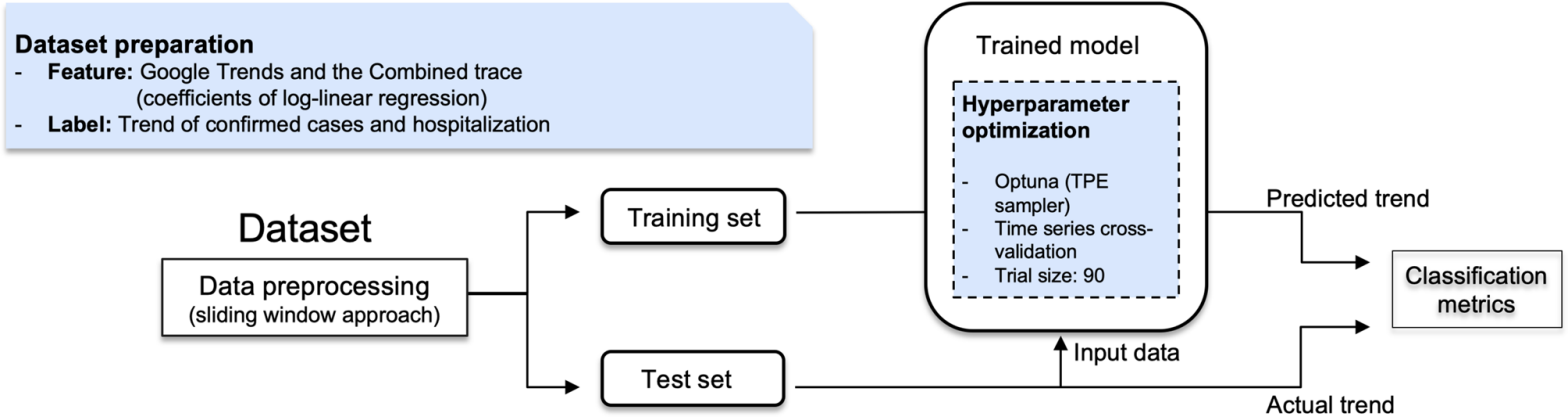
Illustration of the framework for trend forecasting.

The general trend forecasting procedure performed is presented in Fig. 3. Trend forecasting was carried out using the same symptom corpus as during the trend analysis. We first processed the datasets using the sliding window approach, followed by separating into training and testing subsets. Next, we generated Random Forest and LSTM models and evaluated their performance. In order to tune the hyperparameters, time series cross-validation was utilized within the training set. The optimized forecasting model was evaluated on the independent test set by sensitivity, precision, and F1 score.

#### Learning from the time series

As outlined above, a multivariable longitudinal dataset was generated combining the time-stamped slope coefficients of all log-linear regression models, hence reflecting the short-term trends in the original time series data. This data was used to train machine learning models. The time period of the overall dataset ranged from March 2020 to June 2022.

We employed a sliding window approach to collect information on temporal dependence, where the window size was set to 28 days. The sliding window stride was 1 day.

Note that we had previously assigned a time-stamp to each log-linear regression slope coefficient based on the trend observed in the subsequent 14 days. To avoid overoptimism of model predictions we thus assigned a class label to each sliding window according to the type of trend (up-, down-, no-trend) observed in the surveillance data 14 days after the end of the sliding window in digital traces. That means the effective forecasting horizon of our models was set to 14 days.

#### Time series cross-validation

The sktime library (version 0.14.1) provides “SingleWindowSplitter” class to split the training set into temporal folds with constant size. The corresponding training subset of each fold consisted only of observations that occurred before the observation that forms the validation subset. During the time series cross-validation, we set the length of the training period to 90 days/fold. Note that over each of those 90 days we subsequently ran a sliding window to generate training samples, as described earlier. Moreover, we specified the test subsets of 30 days and the stride of 70 days to generate temporal dataset frames.

**Figure 4:**
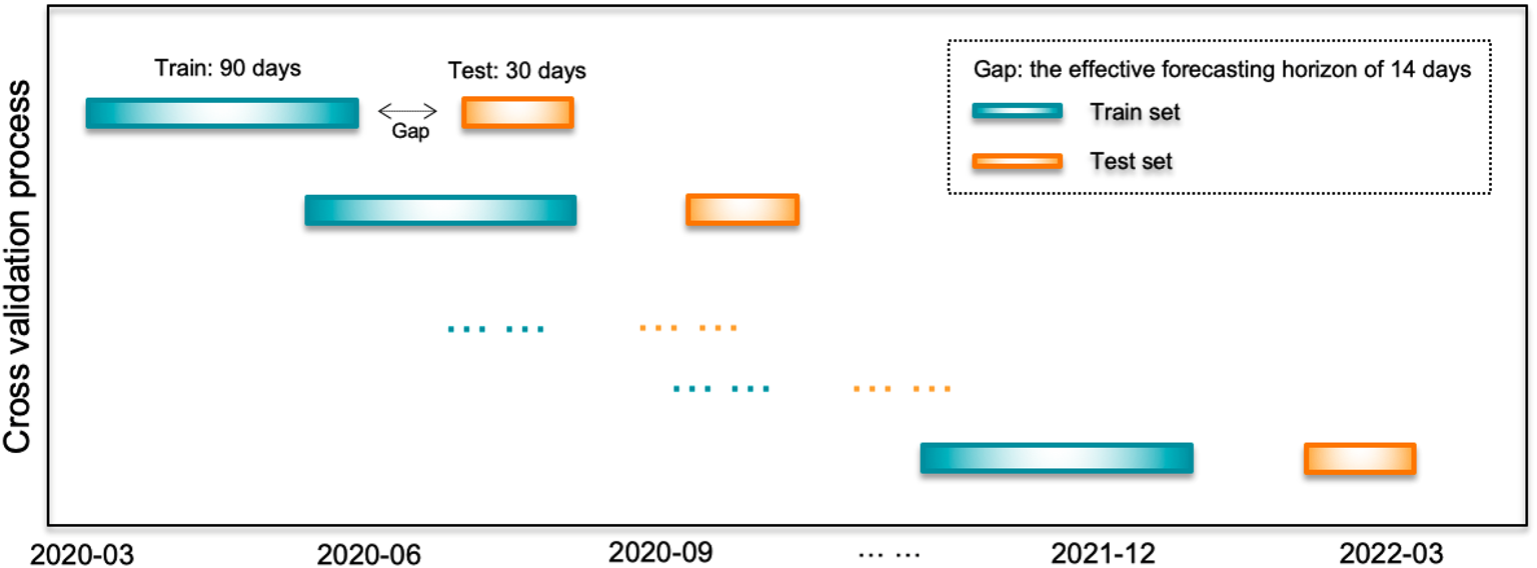
Illustration of time series cross-validation.

We avoided data leakage caused between training and test set by setting a “gap” between them. In this case, the gap was equal to the 14-days sliding window size in log-linear regression model. The following diagram illustrates the concept of time series cross-validation, where the blue observations form the training subsets and the orange observations create the test subsets (Fig. 4).

#### Time split validation

We split the overall time period into training and test sets, i.e. Training set: March 28, 2020 to March 17, 2022; Test set: April 27, 2022 to June 1, 2022. The date here refers to the end of each sliding window, i.e. March 28, 2020: the sliding window from March 1, 2020 to March 28, 2020; March 17, 2022: the sliding window from February 18, 2022 to March 17, 2022; April 27, 2022: the sliding window from March 31, 2022 to April 27, 2022; June 1, 2022: the sliding window from May 5, 2022 to June 1, 2022. The “gap” between training and test sets was equal to the effective forecasting horizon of 14 days as mentioned before.

#### Hyperparameter optimization

Within the time series cross-validation, we automated the process of hyperparameter-tuning, for which we used the Optuna framework (version 2.0.0)^34^.

The range of hyperparameters of Random Forest, including the number of estimators, minimal samples split, max depth range, minimal samples leaf, and maximal number of features, are listed in Supplementary Table S1. For LSTM, we used the Adam optimizer. The other hyperparameters, for instance, the number of epochs, batch size, number of hidden units, number of layers, learning rate and dropout rate, are shown in Supplementary Table S2.

During the hyperparameter tuning, we maximized the validation accuracy for Random Forest and minimize the validation Cross Entropy Loss for LSTM. The Tree-structured Parzen Estimator (TPE)^35^ was defined as sampler. The number of trials was set to 90. Finally, we retrained the model with the suggested hyperparameters and used the hyperparameter-tuned model to conduct the rest of our study.

## Results

### Identification of COVID-19-related symptoms

**Figure 5:**
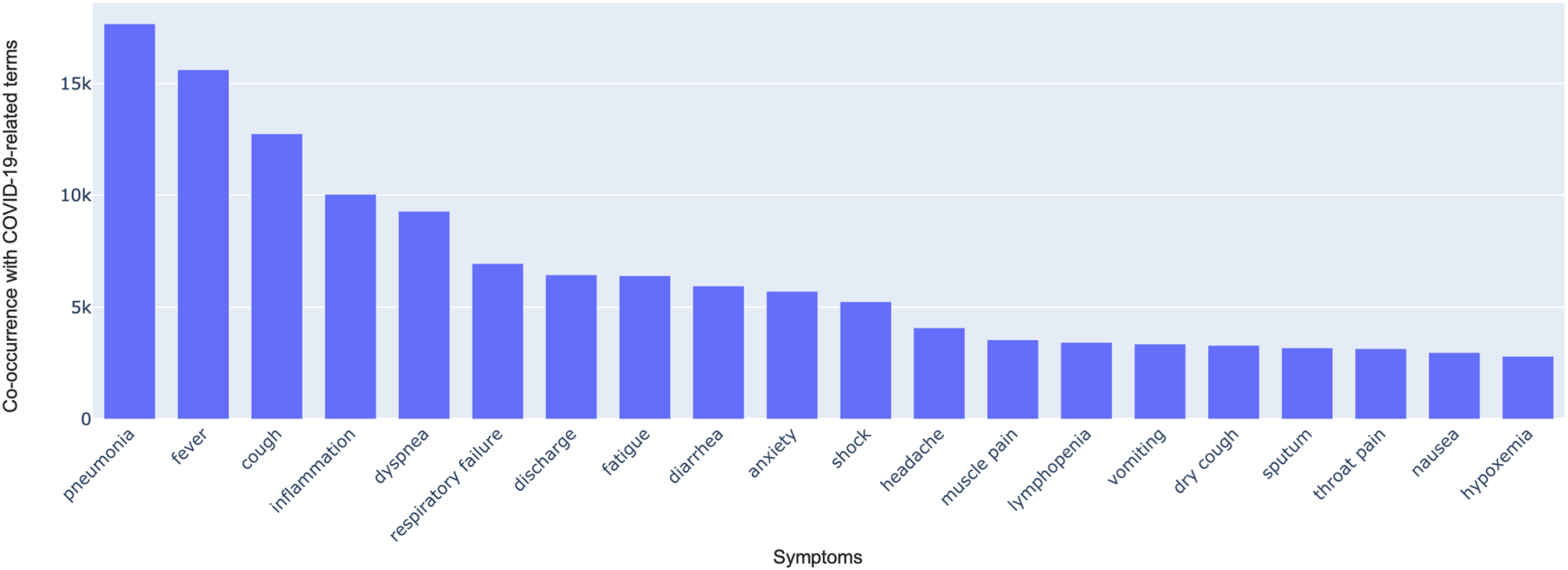
Ranking of the top 20 English symptoms based on the frequency of their co-occurrence with COVID-19 related terms (“COVID”, “Coronavirus disease”, “COVID 19”) in PubMed/PMC. The *x* represents the primary symptom terms with adjusted *P* values less than 0.001, excluding their synonyms.

162 symptoms (249 synonyms) with significant adjusted *P* values (5% significance level) were identified. We ranked the symptom terms based on the frequency of symptom and COVID-19 co-occurrences if they have the same adjusted *P* values. The top 5 most mentioned symptom terms in the COVID-19 related literature were “pneumonia” (17,674, 8.1%, of the total mentioned symptom documents), “fever, pyrexia” (15,617, 7.1%), “cough” (12,756, 5.8%), “inflammation” (10,039, 4.6%), “shortness of breath, dyspnea, breathing difficulty, difficulty breathing, breathlessness, labored respiration” (9278, 4.2%). Figure 5 depicts the top 20 symptoms with their corresponding co-occurrences, which accounting for 61.4% of the total co-occurrences of all identified symptoms.

### Statistical trend analysis

#### Evaluation of trend decomposition

We initially evaluated the applicability of the STL method to forecast the extracted trends from the raw data. Specifically, we utilized the “STL forecast” function from Python’s statsmodels library (version 0.13.5) to extend time series data from an interval [1, *t*] to a future time point $t+k$, represented as $T_\nu (t+k)$ = $T_\nu (1:t)$. The “STL forecast” model was applied to the training data, covering the period from February 2020 to May 2022, and we extrapolated the digital trace time series extending from June 01, 2022, to June 28, 2022. Subsequently, we conducted the STL decomposition on the extrapolated data to derive the corresponding trend component. Through this process, we assessed the correlation between the extrapolated trend and the trend component for the same time period, which was extracted from a STL decomposition of the entire dataset, i.e. training plus test for each of the top 20 symptoms previously listed in Fig. 5 (see Supplementary Table S3 for a complete list and their German translations). The outcomes of this analysis clearly highlighted the robustness of the STL decomposition algorithm with correlations close to 1 (Supplementary Table S4), also in terms of the agreement of significant up-trends in the entire vs. forecasted data for different digital traces (Google Trends, Twitter, and combined of both), see Supplementary Table S5).

#### Symptom-level up-trends in digital traces precede up-trends in surveillance data

Given that our study aims to provide early warning indicators of pandemics in terms of confirmed cases, hospitalization and deaths, we examined whether up-trends in digital traces preceded those in established surveillance data on the level of individual symptoms (Google Trends (168 symptoms), Twitter (204 symptoms)). For this purpose, we first performed a statistical trend analysis in surveillance data from March 2020 to February 2022 as explained in the Methods Section. Then we calculated the sensitivity, precision, and F1 score of each individual symptom based on whether the onsets of up-trends derived from digital traces fell within a 30-day time window ahead of the onsets of up-trends in surveillance data^2^.

Supplementary Table S6 and S7 present the evaluation metrics for the top 20 symptoms from each digital data trace (Google Trends and Twitter) with descending F1 scores for tracking the onsets of up-trends of confirmed cases, deaths, and hospitalization.

The symptom-level up-trend analysis showed that verstopfte Nase (stuffy nose), Gelenkschmerzen (joint pain), Malaise (malaise), laufende Nase (runny nose), and Hautausschlag (skin rash) had strong correlations with up-trends in confirmed cases as evidenced by their F1 scores (0.75, 0.7, 0.7, 0.67, and 0.65), whereas multiples Organversagen (multiple organ failure), Rubor (rubor) and Erbrechen (vomiting) had low F1 scores of 0. Aside from laufende Nase and verstopfte Nase, symptoms like Delirium (delirium), Lethargie (lethargy), and schlechte Ernährung (poor feeding) performed well when tracking both hospitalization and deaths. Interestingly, different symptoms were found with high F1 scores in Google Trends and Twitter.

Overall these findings indicate that certain symptoms mentioned in different digital traces precede up-trends in classical surveillance data and may thus be employed to develop an early alert indicator.

#### Harmonizing multiple symptoms and digital traces into a combined indicator

We next sought to further investigate whether the combination of several symptoms from each digital trace (Google Trends and Twitter) could lead to more accurate detection of up-trends in surveillance data. For this purpose, we focused on the 20 most significant terms according to the hypergeometric test. More specifically, for each symptom ontology, we selected the synonym with the highest search volume in Google Trends and Twitter during the period from February 2020 to February 2022. We then combined these synonyms using the harmonic mean *P*-value (HMP) method^33^. Subsequently, we evaluated the alert detection performance of each digital trace relative to outbreaks in the surveillance data in the same way as described before.

As shown in Table 1, when tracking confirmed cases Google Trends provided an F1 score of 0.5. For Twitter, the F1 score was 0.47. The same metrics to hospitalization and death were considerably weaker around 0.38 or even lower. Furthermore, when harmonizing Google Trends and Twitter into a combined trace, the performance was improved when preceding confirmed cases (an F1 score of 0.59), but only 0.27 when tracking deaths. Based on this, we concluded that (a) digital traces could not be used to construct a reliable early warning indicator for deaths, and (b) a combination of Google Trends and Twitter holds most promises as an early indicator for incident cases and hospitalization. Hence, we focused our subsequent analysis on this combined digital trace.

**Table 1:** Sensitivity, Precision, and F1 Score for different digital traces (Google Trends, Twitter, and combined of both) as an early indicator for an onset of “up-trends” in COVID-19 gold standards (Confirmed cases, Deaths, and Hospitalization).

Gold standard	Google trends			Twitter			Combined		
	Sensitivity	Precision	F1 score	Sensitivity	Precision	F1 score	Sensitivity	Precision	F1 score
Confirmed cases	0.5	0.5	0.5	0.4	0.57	0.47	0.5	0.71	0.59
Deaths	0.5	0.4	0.44	0.25	0.29	0.27	0.25	0.29	0.27
Hospitalization	0.44	0.4	0.42	0.33	0.43	0.38	0.33	0.43	0.38

#### Quantifying the benefit of combined digital trace

**Figure 6:**
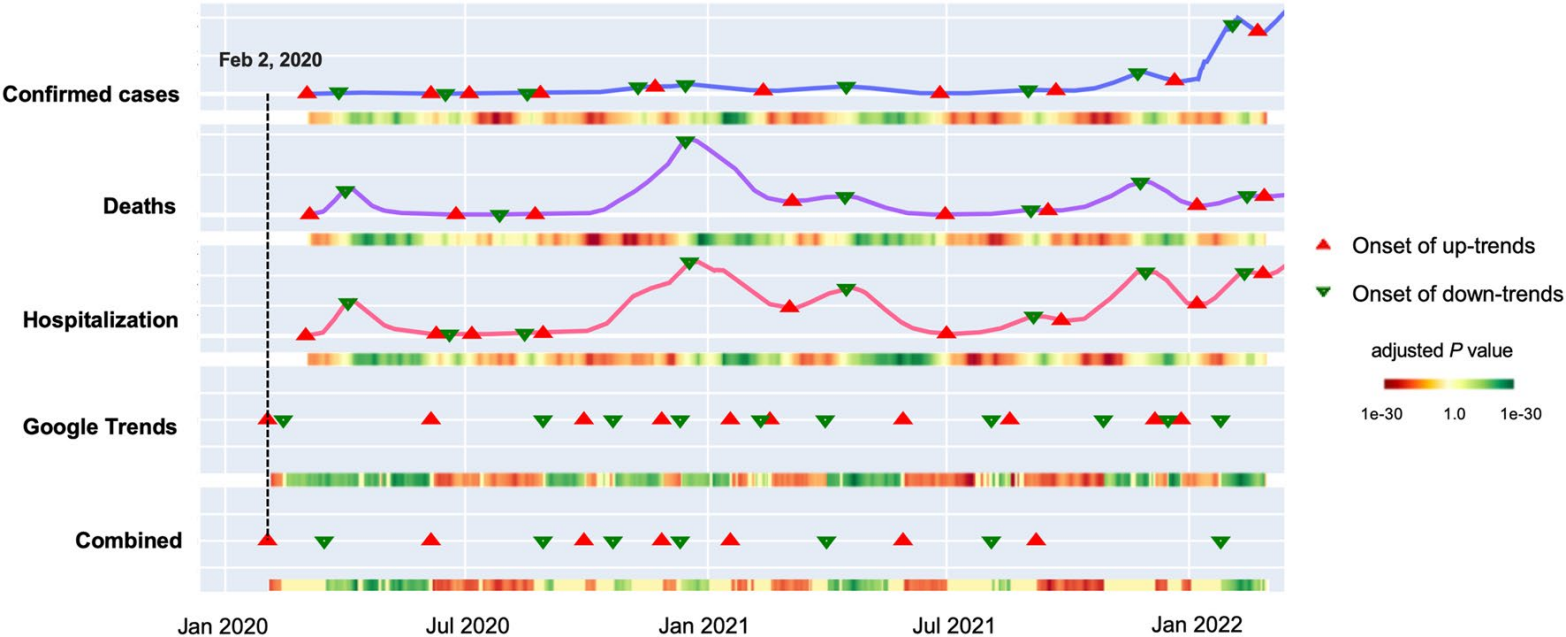
Visualization of the up- and down-trends procedure applied to COVID-19 surveillance, Google Trends, and the combined trace over the period from February 2020 to February 2022. The up- and down-trends were detected by setting a significance level of 5% over the multiple testing corrected *P* values of the log-linear regression model coefficient $\beta$. The adjusted *P* values are shown as colored gradients, where darker red shade signifies the increased confidence of the up-trends, and darker green shade indicates the increased confidence of the down-trends. The minimum adjusted *P* value was 1e−30 for both up- and down-trends. The triangular markers are used to point out the date when the onsets of up- and down-trends were detected based on the significance level. Notably, as shown in the dashed line in the plot, February 2, 2020 was discovered to be an “up-trend” alert date of both traces. The first documented larger outbreak followed a carnival event in the city Heinsberg on February 15, 2020.

**Figure 7:**
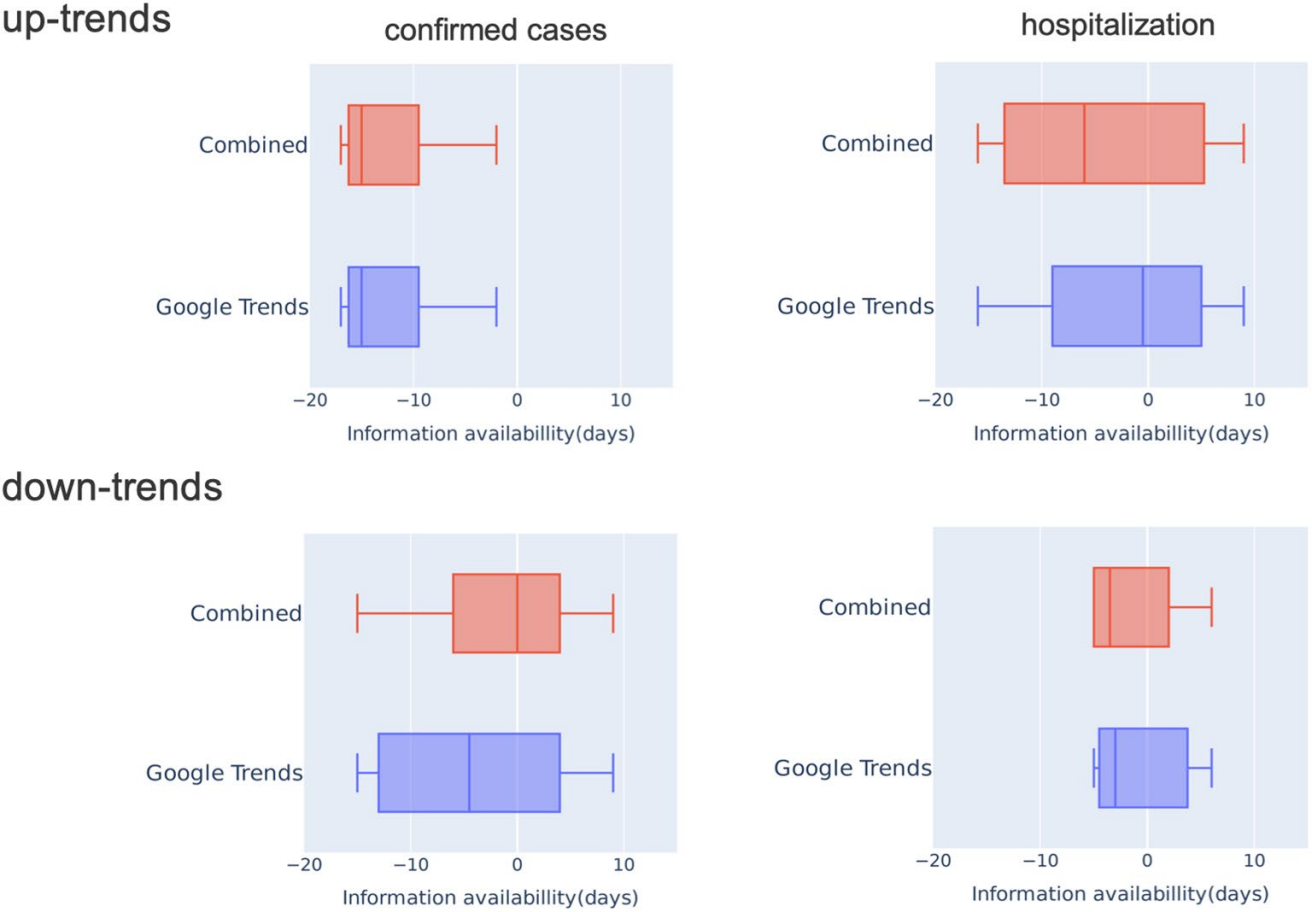
Time lags between significant trends in surveillance data (confirmed cases and hospitalization) relative to significant trends in Google Trends and the combination of Google Trends plus Twitter during the time period February 2020 to February 2022. A negative difference shown on the *x* axis indicates that a significant trend in one of the digital traces preceded the observed trends in confirmed cases and hospitalization, respectively. Median time lag of confirmed cases versus Google Trends: 15 days; confirmed cases vs. Combined trace: 15 days; hospitalization versus Google Trends: 1 day; hospitalization versus Combined trace: 6 day. The number of days have been counted from the end of each sliding window.

To better understand the potential benefit of Google Trends and the combined digital trace as early warning indicators compared to traditional surveillance data it is imperative to quantify the amount of time that trends in both traces precede those in incident cases and hospitalization. For this purpose, we considered the period from February 2, 2020 to February 28, 2022 in digital traces, and counted, how many days the alerts (i.e. the onsets of significant up-trends) in digital traces preceded the first significant up-trends in surveillance data (confirmed cases: March 3, 2020; hospitalization: March 2, 2020; deaths: March 5, 2020) (Fig. 6). Accordingly, we observed that the median time lag between onsets of up-trends in confirmed cases and alerts of Google Trends as well as the combined digital trace was 15 days. The median time lag between hospitalization vs. combined trace was 6 days. For hospitalization vs. Google Trends alone the median time lag was only 1 day (Fig. 7). Noteworthy, these time lags were counted as days from the end of each sliding window.

As depicted in Fig. 7, we conducted a similar analysis for down-trends, emphasizing the alert capabilities of both Google Trends and the combined data trace. For instance, there was a median time lag of 5 days between confirmed cases and Google Trends, while for hospitalization vs. the combined trace, the median time lag was 4 days. Altogether, our analysis shows that trends observed in digital data traces have the potential to serve as an early warning indicator, which could help to save critical time for preparation of governmental counter-measures.

### Trend forecasting via machine learning

Due to the identified statistical association between digital traces, i.e. Google Trends and its combination with Twitter, and surveillance data we next explored, in how far up- and down-trends in the surveillance data could be forecasted via a machine learning classifier, once again using a sliding window approach. Whereas the previously discussed analysis only focused on identifying current trends in digital traces, the aim of such a model was thus to build a predictor for future events. The forecast horizon was set to 14 days, i.e. the aim of the algorithm was to predict the trend in confirmed cases and hospitalization 14 days ahead. It is worth noting that setting any smaller forecast horizon would result into overoptimism, because trends in digital traces had previously been calculated over a 14 days time period via the log-linear regression model, and in reality we cannot forecast trends less than 14 days ahead. The setting of forecasting horizon specifically applies to the last time point in each sliding window.

Table 2 summarizes the sensitivity, precision, and F1 scores of Random Forest and LSTM models for predicting trends of confirmed cases and hospitalization (from end of March 2022 to June 2022). During the training procedure, we performed a 9-fold time series cross-validation scheme to tune hyperparameters. The suggested hyperparameter values of the best-performing Random Forest and LSTM models are shown in Supplementary Table S8. The result indicates a better prediction performance of the LSTM model, which reached an F1 of 0.98 for up-trend forecasting of confirmed case and an F1 score of 0.97 for up-trend forecasting of hospitalization using the combination of Google Trends plus Twitter (2b and 2d). Besides, a down-trend in confirmed cases as well as hospitalizations was equally well predicted by F1 scores of 0.91 and 0.96, respectively. Considering all three possible classes (up-trend, down-trend, no-trend) a weighted average F1 of 0.95 for confirmed cases and 0.94 for hospitalization could be achieved.

**Table 2:** Random Forest and LSTM performances for trend forecasting: Models were built on Google Trends alone (a, c) and the combination of Google Trends and Twitter (b, d).

Model	Metrics	Up-trend	Down-trend	Macro avg.	Weighted avg.
(a) Google Trends-Confirmed cases					
Random forest	Sensitivity	1	0.33	0.44	0.72
	Precision	0.71	1	0.57	0.64
	F1 score	0.83	0.5	0.44	0.64
LSTM	Sensitivity	1	0.5	0.72	0.86
	Precision	0.92	1	0.83	0.88
	F1 score	0.96	0.67	0.75	0.85
(b) Combined-Confirmed cases					
Random forest	Sensitivity	0.92	1	0.64	0.78
	Precision	1	0.43	0.48	0.74
	F1 score	0.96	0.6	0.52	0.74
LSTM	Sensitivity	0.96	0.83	0.93	0.94
	Precision	1	1	0.92	0.96
	F1 score	0.98	0.91	0.91	0.95
(c) Google trends-hospitalization					
Random forest	Sensitivity	1	0.75	0.58	0.75
	Precision	0.67	1	0.56	0.67
	F1 score	0.8	0.86	0.55	0.69
LSTM	Sensitivity	0.89	0.58	0.82	0.81
	Precision	1	1	0.82	0.91
	F1 score	0.94	0.74	0.77	0.82
(d) Combined-hospitalization					
Random forest	Sensitivity	1	1	0.67	0.83
	Precision	0.9	0.75	0.55	0.70
	F1 score	0.95	0.86	0.60	0.76
LSTM	Sensitivity	1	0.92	0.92	0.94
	Precision	0.95	1	0.93	0.95
	F1 score	0.97	0.96	0.92	0.94

All models were tested during the out-of-sample period from end of March to June 2022.

#### Interpretation of trend forecasting model

**Figure 8:**
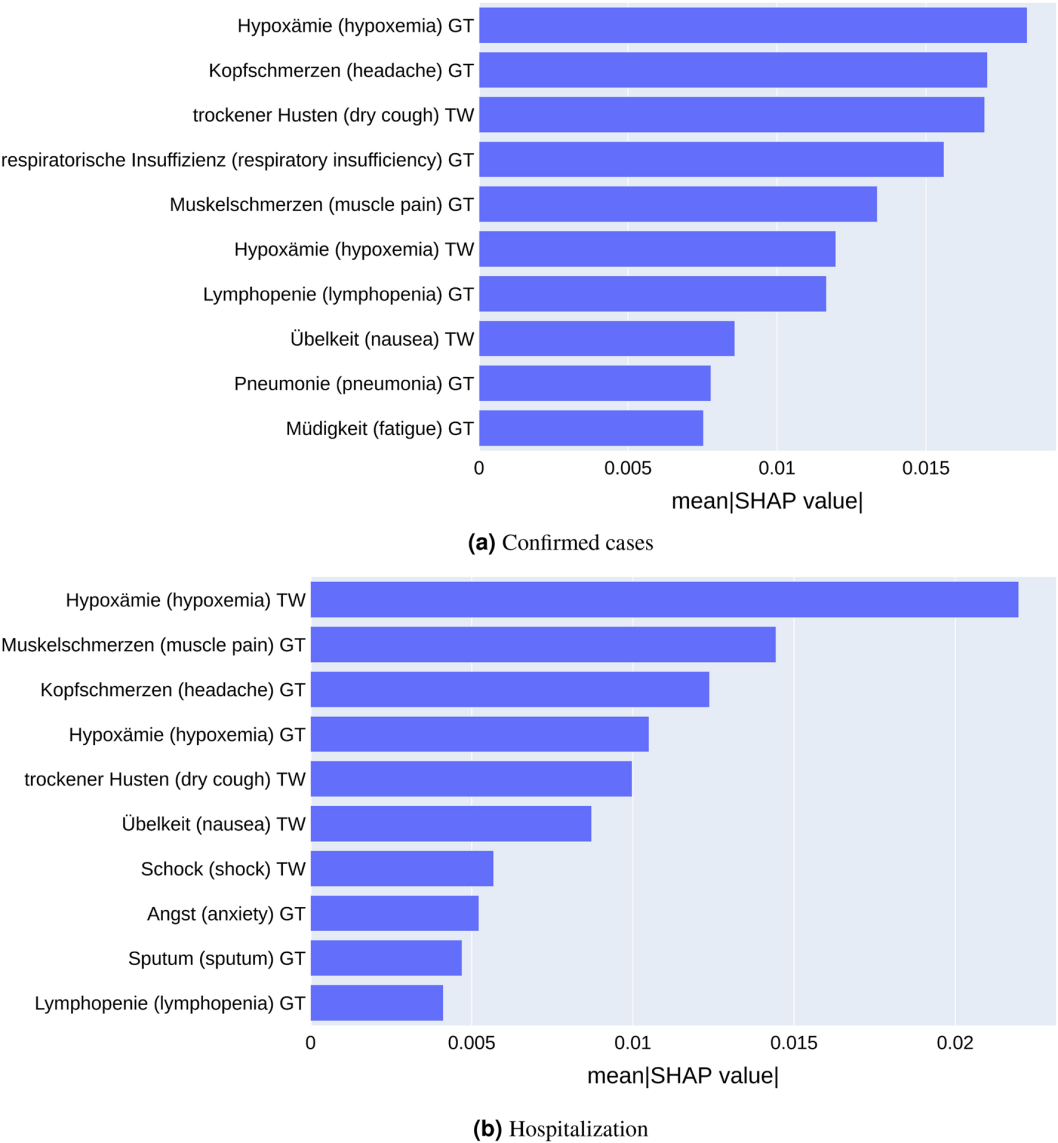
SHAP values calculated for LSTM models predicting confirmed incident cases (**a**) and hospitalization (**b**), respectively. Models have been trained on combined Google Trends (GT) plus Twitter (TW) data. Only mean SHAP values for predicting up-trends are shown.

We performed an analysis of the best performing LSTM models for predicting incident cases as well as hospitalization via Shapley Additive Explanations (SHAP)^36^ to understand the influence of individual Google search and Twitter terms for model predictions of up-trends. Figure 8a and  b depict the bar plots of predictive symptoms with their descending mean absolute SHAP values. For instance, Hypoxämie (hypoxemia) from Google Trends & Twitter, Kopfschmerzen (headache) and Muskelschmerzen (muscle pain) from Google Trends, trockener Husten (dry cough) and Übelkeit (nausea) from Twitter were indicative of up-trends in confirmed cases and hospitalization. Moreover, respiratorische Insuffizienz (respiratory insufficiency), Pneumonie (pneumonia) and Müdigkeit (fatigue) from Google Trends were relevant symptom for forecasting up-trends in confirmed cases. The same pattern was observed in Schock (shock) (Twitter), Angst (anxiety) and Sputum (sputum) (Google Trends) for predicting hospitalization up-trends. These findings are generally in line with the previously presented statistical trend analysis of individual symptoms.

## Discussion

This work explored the possibility to use digital traces, in particular Google Trends and Twitter, in Germany to develop an early alert indicator and trend forecasting model. Existing work going into a similar direction has previously only been published for countries other than Germany^2,37–46^. Since the use of social media and internet searches is likely influenced by socio-economic and cultural background, we believe that a careful evaluation of according approaches for different regions of the world is necessary, and in this sense our work fills a gap. Furthermore, existing articles only focused on a limited set of ad-hoc query terms without a systematic way to identify them based on automated literature mining, as performed in our work. In contrast, our work resulted into a complete COVID-19-symptom corpus.

After transforming the mentioning of symptoms into a time series, we came up with a consensus indicator using log-linear regression, which we statistically evaluated against observed trends in gold standard surveillance data. We discovered that Google Trends and the combination with Twitter demonstrated a significant correlation with up-trends in surveillance data. These findings are consistent with the conclusion of the study by Kogan et al. that harmonizing digital data traces could anticipate changes in COVID-19 related surveillance data^2^. In light of the strength of the statistical association and the observed time lag between digital traces and surveillance data, we further examined the ability to forecast future trends in the surveillance data. Here, we built, evaluated, and compared Random Forests and LSTM models, leading to the following findings: (1) The models built on a combination of Google Trends and Twitter outperformed those that were built only using Google Trends. (2) LSTMs in most cases outperformed Random Forests. (3) In comparison to the previous study by Kogan et al.^2^, which achieved a sensitivity of 0.75 for anticipating COVID-19 infection up-trends in the USA using an Bayesian model 1 week in advance, our LSTM model showed F1 scores of 0.98 and 0.97 for confirmed cases and hospitalization in Germany with a larger forecast horizon of 14 days. This demonstrates the potential of modern machine learning algorithms for trend forecasting based on digital traces, which other studies did not explore to the same extent so far. (4) Some symptoms, like Hypoxämie (hypoxemia), Kopfschmerzen (headache), Muskelschmerzen (muscle pain), trockener Husten (dry cough), and respiratorische Insuffizienz (respiratory insufficiency) played important roles in predicting surveillance up-trends. Hypoxemia is a symptom typically observed in severe COVID-19 cases, which is a sign of respiratory insufficiency^47^. An increase of Google searches and tweets of these symptoms could thus indicate a general up-trend of severe cases, which is itself associated with an increasing rate of hospitalization. Headache, muscle pain, and dry cough are general symptoms of COVID-19 infection. An increase of internet searches and tweets is thus an indicator of a general up-trend of incident cases.

While our work provides valuable insights into trend analysis and forecasting models to track pandemic situations such as COVID-19, there are several limitations that need to be mentioned. Firstly, surveillance data is principally limited due to under-reporting, i.e. the true number of infected cases is most likely higher than the observed one, and this could potentially influence our trend analysis and trend forecasting results in an unknown way. Secondly, digital traces and specifically social media data likely suffer from a principal bias towards younger age groups and people with higher education level.

Our ML models focused on predicting the trend (up, down, no-trend) in classical surveillance data 14 days ahead. We believe that this is relevant, because it could provide decision makers with a time window to potentially take countermeasures, e.g. non-pharmaceutical interventions. Future work could address further relevant forecasting questions, such as predicting the length of a trend, or the time dependent risk to switch to an up-trend. Whilst the framework presented here should be generally applicable to other infectious diseases (e.g. influenza or RSV) the according empirical evaluation has to be referred to future work as well.

## Conclusion

To our knowledge this is the first study to analyse and model COVID-19 related digital traces derived from a systematic literature text mining approach in Germany. Our work specifically demonstrates the potential of combining Google Trends and Twitter data to derive an early warning indicator and to accurately forecast trends in standard surveillance data two weeks in advance. Systematic tracking of digital traces could in the future complement other approaches, including more established surveillance data assessment, mobility data mining and text mining of news articles in order to react earlier to future pandemic situations in Germany. Our work in this regard can be seen as a first step towards establishing an accurate and comprehensive early warning system.

### Supplementary Information

Supplementary Tables.

## Data Availability

Symptom Ontology data can be retrieved from EBI Symptom Ontology (https://www.ebi.ac.uk/ols/ontologies/symp), Surveillance data can be retrieved from Robert Koch-Institut (RKI) GitHub repository (https://github.com/orgs/robert-koch-institut/repositories), and digital trace data can be retrieved via the corresponding API tools. The data needed to evaluate the results are presented in the paper or supplementary documents. The code of the model is publicly available at https://github.com/danqi123/pandemic_alert_model_social_media.
