# Supplementary material for: Development of an early alert model for pandemic situations in Germany
Source: Sci Rep. 2023 Nov 27;13:20780. doi: 10.1038/s41598-023-48096-3 (PMC10682010; doi:10.1038/s41598-023-48096-3)
Supplement: Supplementary file 1 — Supplementary Tables. [file 41598_2023_48096_MOESM1_ESM.pdf]

Supplementary Material: Development of an early alert model for pandemic situations in Germany

Danqi Wang[1,*], Manuel Lentzen[1,2], Jonas Botz[1,2], Diego Valderrama[1,2], Lucille Deplante[3], Jules Perrio[3], Marie Génin[3], Edward Thommes[4], Laurent Coudeville[4], and Holger Fröh-lich[1,2*]

1Department of Bioinformatics, Fraunhofer Institute for Algorithms and Scientific Computing (SCAI), Schloss Birlinghoven, 53757 Sankt Augustin, Germany
2Bonn-Aachen International Center for IT, University of Bonn, Friedrich Hirzebruch-Allee 6, 53115 Bonn, Germany
3Quinten Health, 8 rue vernier, 75017 Paris, France
4Sanofi, Paris, France
*danqi.wang@scai.fraunhofer.de, holger.froehlich@scai.fraunhofer.de

Hyperparameters	Range
n_estimators	suggest_categorical ("n_estimators", [200, 300, 400, 500, 600])
min_samples_split	suggest_categorical ("min_samples_split", [2, 5, 10])
max_depth	suggest_int ("max_depth", 5, 50, step=10)
min_samples_leaf	suggest_categorical ("min_samples_leaf", [1, 2, 4])
max_features	suggest_categorical ("max_features", ["log2", "sqrt"])

Supplementary Table S1. Overview of hyperparameters tuning in Random Forest

Hyperparameters	Range
num_epochs	suggest_categorical ("num_epochs", [400, 500, 600, 700])
batch_size	suggest_categorical ("batch_size", [16, 32, 64])
n_hidden	suggest_int ("n_hidden", 60, 120)
n_layers	suggest_int ("n_layers", 2, 4)
lr	suggest_loguniform ("lr", 4e-4, 2e-3)
dropout	suggest_float ("dropout", 0, 0.2, step=0.1)

Supplementary Table S2. Overview of hyperparameters tuning in LSTMs

German	English
Lymphopenie, Müdigkeit, Rachenschmerzen (Halsentzündung), Sputum, Muskelschmerzen, Entzüdung, Erbrechen, Ausfluss, Fieber, trockener Husten, Schock, Husten, Kopfschmerzen, Angst, respiratorische Insuffizienz, Übelkeit, Dyspnoe (Atemnot), Pneumonie (Lungenentzüdung), Hypoxämie, Durchfall	lymphopenia, fatigue, throat pain (sore throat), sputum, muscle pain, inflammation, vomiting, discharge, fever, dry cough, shock, cough, headache, anxiety, respiratory insufficiency, nausea, dyspnea (breathing difficulty), pneumonia, hypoxemia, diarrhea

Supplementary Table S3. The top 20 German symptom terms, identified using the hypergeometric test, were utilized in generating the combined Google Trends and Twitter indicator. Synonyms for Twitter terms are provided in parentheses. English translations are also presented in the table for reference.

**Symptom**	**Pearson correlation coefficient**	***P* value**
Durchfall	0.9998005555917560	1.00e-45
Angst	0.9981556556212010	3.59e-33
Rachenschmerzen	0.9999407792811380	1.40e-52
Fieber	0.9999274355963450	1.96e-51
Entzündung	0.9740894119438370	2.61e-18
Muskelschmerzen	0.9998760682521950	2.06e-48
Schock	0.9998590228815750	1.10e-47
trockener Husten	0.9998429002133480	4.50e-47
Sputum	0.9940164041781680	1.55e-26
Husten	0.9999869433534100	4.07e-61
Kopfschmerzen	0.9997930536930420	1.62e-45
Erbrechen	0.9991635929985660	1.24e-37
Übelkeit	0.9999934053773420	5.66e-65
Lymphopenie	0.9999234391500330	3.94e-51
Pneumonie	0.9984700197755410	3.17e-34
respiratorische Insuffizienz	0.9971986668536020	8.18e-31
Dyspnoe	0.9995486620453160	4.08e-41
Hypoxämie	0.999886707561521	6.43e-49
Müdigkeit	0.9309945673760730	6.91e-13
Ausfluss	0.9566952576306800	1.87e-15

(a) Google Trends

Symptom	Pearson correlation coefficient	*P* value
Husten	0.9997291175665880	5.36e-44
Ausfluss	0.9960485657209360	7.11e-29
Übelkeit	0.999493234779096	1.84e-40
Schock	0.9982325979665140	2.06e-33
Hypoxämie	0.9988365370340710	9.03e-36
Erbrechen	0.9163027134219330	7.81e-12
Muskelschmerzen	0.9999209246080370	6.00e-51
Durchfall	0.9989454785926960	2.52e-36
Lungenentzündung	0.9998877682372970	5.69e-49
Halsentzündung	0.9953103017258910	6.56e-28
Fieber	0.9997289933202190	5.39e-44
Kopfschmerzen	0.9994254982302790	9.40e-40
Lymphopenie	0.741891560168267	6.23e-06
Entzündung	0.9999124660110680	2.25e-50
trockener Husten	0.9998457196127890	3.56e-47
Atemnot	0.9978499098652420	2.63e-32
Sputum	0.995720663200947	2.00e-28
Müdigkeit	0.8960527017950110	1.16e-10
respiratorische Insuffizienz	0.9994065081340140	1.43e-39
Angst	0.9624300283284250	3.05e-16

(b) Twitter

Supplemental Table S4. a) Google Trends & b) Twitter symptom-level trend component correlation between extrapolated trend component and the trend, which was extracted from an STL decomposition of the entire dataset (February 01, 2020, to June 28, 2022). The extrapolated time period was from June 01, 2022 to June 28, 2022. The symptom terms mentioned in the table represent the top 20 most significant terms from the hypergeometric test.

Proxy	Sensitivity	Precision	F1 score
Google Trends	0.92	1	0.96
Twitter	0.9	1	0.95
Combined	0.89	1	0.94

Supplemental Table S5. The evaluation metrics (Sensitivity, Precision, and F1 score) for the onsets of "up-trends" generated from STL-forecasted digital traces (Google Trends, Twitter, and the combination of both) compared to the onsets of "up-trends" generated from the original entire dataset. The STL-forecasted period was from June 01, 2022, to June 28, 2022. A "true-positive" (TP) event was declared if an "up-trend" in the forecasted data source (i.e. trend component) fell within a 30-day window of the onset of 'up-trends' detected in the original dataset.

German symptom	English	sensitivity	precision	F1 score
verstopfte Nase	stuffy nose	0.7	0.8	0.75
Gelenkschmerzen	joint pain	0.7	0.7	0.7
Malaise	malaise	0.8	0.62	0.7
laufende Nase	runny nose	0.6	0.75	0.67
Hautausschlag	skin rash	0.7	0.6	0.65
Nesselsucht	hives	0.6	0.67	0.63
hämatogen	hematogenous	0.5	0.83	0.63
Lethargie	lethargy	0.6	0.64	0.62
Fehlgeburt	miscarriage	0.6	0.6	0.6
Erschöpfung	exhaustion	0.5	0.63	0.56
Schüttelfrost	chills	0.5	0.63	0.56
trockener Husten	dry cough	0.5	0.63	0.56
Muskelschmerzen	muscle pain	0.5	0.63	0.56
Delirium	delirium	0.6	0.5	0.55
Rachenschmerzen	throat pain	0.4	0.8	0.53
Wassersucht	dropsy	0.4	0.8	0.53
Hautrötung	skin redness	0.4	0.8	0.53
Myalgie	myalgia	0.5	0.56	0.53
Gliederschmerzen	body ache	0.5	0.56	0.53
septischer Schock	septic shock	0.5	0.56	0.53

(a) Confirmed cases

German symptom	English	sensitivity	precision	F1 score
laufende Nase	runny nose	0.88	0.88	0.88
Delirium	delirium	0.88	0.58	0.7
Lethargie	lethargy	0.75	0.64	0.69
schlechte Ernährung	poor feeding	0.5	1	0.67
verstopfte Nase	stuffy nose	0.75	0.6	0.67
Thrombozytopenie	thrombocytopenia	0.63	0.63	0.63
Sputum	sputum	0.63	0.63	0.63
septischer Schock	septic shock	0.63	0.56	0.59
Fieber	fever	0.63	0.56	0.59
hämatogen	hematogenous	0.5	0.67	0.57
Arrhythmie	arrhythmia	0.5	0.63	0.56
Sinustachykardie	sinus tachycardia	0.63	0.5	0.56
Urtikaria	urticaria	0.63	0.5	0.56

Pneumonie	pneumonia	0.63	0.45	0.53
Verwirrung	confusion	0.63	0.45	0.53
Keuchen	wheezing	0.63	0.45	0.53
Schleim	phlegm	0.63	0.45	0.53
Erschöpfung	exhaustion	0.5	0.5	0.5
Bronchitis	bronchitis	0.5	0.5	0.5
Malaise	myalgia	0.63	0.38	0.48

(b) Deaths

German symptom	English	sensitivity	precision	F1 score
laufende Nase	runny nose	0.67	0.75	0.71
Delirium	delirium	0.78	0.58	0.67
Gliederschmerzen	body ache	0.67	0.67	0.67
Lethargie	lethargy	0.67	0.64	0.65
schlechte Ernährung	poor feeding	0.44	1	0.62
Pneumonie	pneumonia	0.67	0.55	0.6
Verwirrung	confusion	0.67	0.55	0.6
Keuchen	wheezing	0.67	0.55	0.6
Schleim	phlegm	0.67	0.55	0.6
verstopfte Nase	stuffy nose	0.56	0.6	0.58
septischer Schock	septic shock	0.56	0.56	0.56
Schock	shock	0.56	0.56	0.56
Knistern	crackles	0.56	0.56	0.56
Durchfall	diarrhoea	0.56	0.56	0.56
Malaise	myalgia	0.67	0.46	0.55
Lungenödem	pulmonary edema	0.67	0.46	0.55
hämatogen	hematogenous	0.44	0.67	0.53
Dyspnoe	dyspnea	0.56	0.5	0.53
Bronchitis	bronchitis	0.56	0.5	0.53
Sinustachykardie	sinus tachycardia	0.56	0.5	0.53

(c) Hospitalization

Supplementary Table S6 Sensitivity, Precision, and F1 Score for top 20 symptoms with descending F1 scores from Google Trends as an early indicator for an onset of “up-trends” in COVID-19 gold standards (a) Confirmed cases (b) Deaths (c) Hospitalization.

German symptom	English	sensitivity	precision	F1 score
Steifheit	rigors	0.8	0.89	0.84
Schlaganfall	stroke	0.6	1	0.75
Erschöpfung	exhaustion	0.6	0.86	0.71
Diarrhö	diarrhea	0.7	0.64	0.67
Hirnschlag	brain attack	0.6	0.75	0.67
Reizbarkeit	irritability	0.7	0.58	0.64
schwere Lungenentzündung	severe pneumonia	0.6	0.67	0.63
Entzündung	inflammation	0.5	0.83	0.63
Enzephalitis	encephalitis	0.5	0.83	0.63
Halsentzündung	sore throat	0.6	0.6	0.6
Nierenversagen	renal failure	0.5	0.71	0.59
laufende Nase	runny nose	0.5	0.71	0.59
Meningoenzephalitis	meningoencephalitis	0.5	0.71	0.59
Verwirrung	confusion	0.5	0.71	0.59
hohes Fieber	high fever	0.5	0.71	0.59
Nesselsucht	hives	0.6	0.55	0.57
trockener Husten	dry cough	0.5	0.63	0.56
Hirninfarkt	cerebral accident	0.5	0.63	0.56
Husten	cough	0.5	0.63	0.56
Schnupfen	rhinitis	0.5	0.63	0.56

(a) Confirmed cases

German symptom	English	sensitivity	precision	F1 score
Erschöpfung	exhaustion	0.63	0.71	0.67
Nierenversagen	renal failure	0.63	0.71	0.67
Pharyngitis	pharyngitis	0.63	0.71	0.67
Verwirrung	confusion	0.63	0.71	0.67
Diarrhö	diarrhea	0.75	0.55	0.63
Hypoventilation	hypoventilation	0.5	0.8	0.62
Durchfall	diarrhoea	0.63	0.56	0.59
Müdigkeit	fatigue	0.5	0.67	0.57
erschwerte Atmung	labored respiration	0.5	0.67	0.57
leichte Bindehautentzündung	mild conjunctivitis	0.38	0.75	0.5
Koagulopathie	coagulopathy	0.38	0.75	0.5
Ödeme in der Lunge	oedematous lungs	0.38	0.75	0.5
Epistaxis	epistaxis	0.5	0.5	0.5

Benommenheit	dizziness	0.5	0.5	0.5
Orthopnoe	orthopnea	0.5	0.5	0.5
mangelnde Koordination	lack of coordination	0.5	0.5	0.5
Steifheit	rigors	0.5	0.44	0.47
Atemprobleme	breathing problems	0.5	0.44	0.47
Gelenkschmerzen	joint pain	0.5	0.44	0.47
Enanthem	enanthem	0.38	0.6	0.46

(b) Deaths

**German symptom**	**English**	**sensitivity**	**precision**	**F1 score**
Steifheit	rigors	0.67	0.67	0.67
Benommenheit	dizziness	0.67	0.63	0.65
verstopfte Nase	stuffy nose	0.67	0.6	0.63
Keuchen	wheezing	0.67	0.6	0.63
schlechte Ernährung	poor feeding	0.67	0.6	0.63
Erschöpfung	exhaustion	0.56	0.71	0.63
Nierenversagen	renal failure	0.56	0.71	0.63
Depression	depression	0.56	0.71	0.63
leichte Bindehautentzündung	mild conjunctivitis	0.44	1	0.62
Entzündung	inflammation	0.56	0.67	0.61
Diarrhö	diarrhea	0.67	0.55	0.6
Hypoventilation	hypoventilation	0.44	0.8	0.57
Durchfall	diarrhoea	0.56	0.56	0.56
Schwäche	weakness	0.56	0.56	0.56
schwere Lungenentzündung	severe pneumonia	0.56	0.56	0.56
Appetitlosigkeit	loss of appetite	0.56	0.55	0.55
erschwerte Atmung	labored respiration	0.44	0.67	0.53
Enzephalitis	encephalitis	0.44	0.67	0.53
Gliederschmerzen	body ache	0.44	0.57	0.5
Bronchitis	bronchitis	0.44	0.57	0.5

(c) Hospitalization

Supplementary Table S7 Sensitivity, Precision, and F1 Score for top 20 symptoms with descending F1 scores from Twitter as an early indicator for an onset of “up-trends” in COVID-19 gold standards (a) Confirmed cases (b) Deaths (c) Hospitalization.

Hyperparameters	Google Trends-Confirmed cases	Combined-Confirmed cases	Google Trends-Hospitalization	Combined-Hospitalization
n_estimators	600	300	300	200
min_samples_split	2	2	5	2
max_depth	15	25	25	35
min_samples_leaf	4	2	2	1
max_features	“log2”	“log2”	“log2”	“log2”

(a) Optimal Random Forest hyperparameters

Hyperparameters	Google Trends-Confirmed cases	Combined-Confirmed cases	Google Trends-Hospitalization	Combined-Hospitalization
num_epochs	700	500	600	400
batch_size	16	64	64	64
n_hidden	65	115	75	85
n_layers	3	3	3	3
lr	0.00118268283736384	0.0009780464862646773	0.0004339449402018055	0.001249464512293463
dropout	0.1	0.1	0.1	0.1

(b) Optimal LSTMs hyperparameters

Supplementary Table S8. Optimal hyperparameters in (a) Random Forest and (b) LSTMs for forecasting trends of confirmed cases and hospitalization.
